# Tracking Migrating T Cells in Real Time

**DOI:** 10.1371/journal.pbio.0030205

**Published:** 2005-05-03

Ever since Robert Hooke startled the world with finely rendered illustrations of “minute bodies” in his 1665 book, Micrographia, our understanding of the microscopic world within and around us has mushroomed with each technological advance. Though imaging capability has developed light-years beyond the compound microscope that inspired Hooke's cork cell epiphany, biologists have only recently been able to observe living cells in chunks of tissue extracted from an organism—an approach that's critical for studying processes like cell differentiation and development.

Ellen Robey and her group study the mechanisms of cell differentiation and cell fate by tracking T cell development in mouse models. In a new study, Colleen Witt, Ellen Robey, and their colleagues take advantage of a recent innovation called two-photon microscopy to visualize the migration of developing T cells, or thymocytes, in intact thymuses extracted from mice. They find that after cells undergo positive selection—which seals their fate as either helper T or killer T cells—they make a beeline for the thymus interior (called the medulla). Though it's been known that positively selected thymocytes migrate to the medulla, this study shows that migration follows a clear directional course, possibly guided by long-range signaling cues.[Fig pbio-0030205-g001]

**Figure pbio-0030205-g001:**
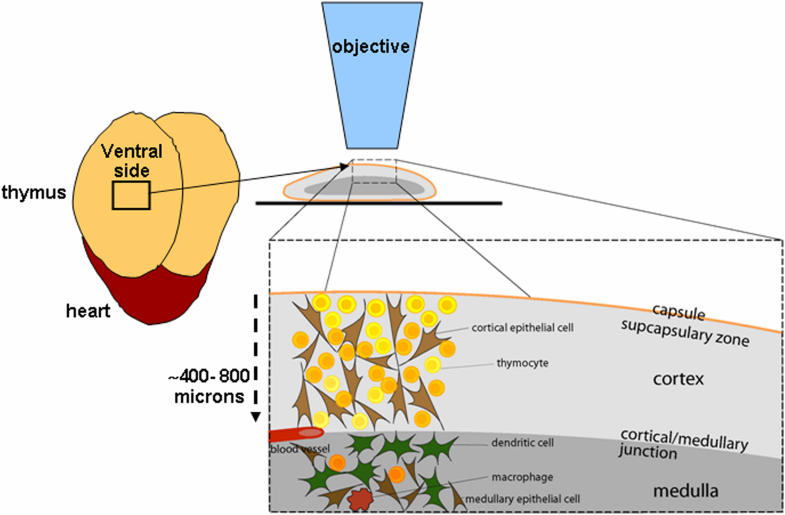
Real-time visualization of thymocytes within intact thymic lobes using two-photon microscopy

Because two-photon microscopy can penetrate tissue at high resolution without distorting or damaging the specimen, the authors could characterize thymocytes moving through their native tissue environment and interacting with the molecules and cells they would normally encounter. (For more on two-photon microscopy, see the Primer by David Piston [10.1371/journal.pbio.0030207] and the “Tracking the Details of an Immune Cell Rendezvous in 3-D” [DOI: 10.1371/journal.pbio.0030206].)

In the service of optimum immune defense, the mammalian adaptive immune system churns out billions of T cells a day. Precursor T cells originate in the bone marrow and migrate to the thymus, where their immune mettle is tested by a selection process that only about 1% will survive. Immature double-positive thymocytes—so-called because they have the protein markers associated with both helper (CD4) and killer (CD8) T cells—inhabit the outer thymic layer, called the cortex, while single-positive thymocytes—which have lost either the CD4 or CD8 marker following positive selection—are found in the central medulla. How a thymocyte reacts to other lymphocytes as it wends its way through the thymus determines whether it undergoes positive selection and matures into a helper or killer T cell or undergoes negative selection and programmed cell death. The signaling cues that guide this process remain obscure.

Witt et al. engineered mice with thymocytes tagged with green fluorescent protein (GFP), removed their thymic lobes for microscopic analysis when they were 4.5 to 5.5 weeks old, then observed the behavior of the glowing cells. The GFP cells in the cortex showed distinct differences in motility, morphology, and migratory behavior: low-motility cells had a spherical, nonpolar shape, moved with a modestly protruding leading edge, and sometimes paused; high-motility cells had a clear leading edge that moved in fits and starts and never paused. Once on the move, high-motility cells mostly hewed to a single direction while low motility cells often retraced their steps. Unlike the low-motility cells, the high-motility cells traveled in a linear manner through the cortex, suggesting directed migration.

Since there were so few of the fast-moving, inwardly migrating cells, the authors hypothesized that they had undergone positive selection—which they went on to confirm in transgenic mouse models. From these results, Witt et al. conclude that positive selection triggers a “rapid directional migration pattern.” And because that migration corresponds to an area of the cortex that extends up to 200 microns below the outer layer of the thymus, it appears to be guided by long-range signaling cues.

As often happens in biology, close observation of a process reveals more complexity and raises more questions about the mechanics underlying it. Homing in on the source of these long-range signaling cues and characterizing the migratory patterns of the large number of slow-moving cells will go a long way toward understanding how the major components of immunity acquire their defensive chops.

